# Intratumoural Delivery of mRNA Loaded on a Cationic Hyper-Branched Cyclodextrin-Based Polymer Induced an Anti-Tumour Immunological Response in Melanoma

**DOI:** 10.3390/cancers15143748

**Published:** 2023-07-24

**Authors:** Yousef Khazaei Monfared, Mohammad Mahmoudian, Parvin Zakeri-Milani, Claudio Cecone, Tomoya Hayashi, Ken J. Ishii, João Conde, Adrián Matencio, Francesco Trotta

**Affiliations:** 1 Department of Chemistry, University of Turin, 10125 Turin, Italy; yousef.khazaeimonfared@unito.it (Y.K.M.); claudio.cecone@unito.it (C.C.); adrian.matencioduran@unito.it (A.M.); 2 Faculty of Pharmacy, Tabriz University of Medical Sciences, Tabriz 5165665931, Iran; mahmoodian.nano@gmail.com; 3 Division of Vaccine Science, Department of Microbiology and Immunology, The Institute of Medical Science, The University of Tokyo (IMSUT), Tokyo 113-8654, Japan; tomoya-h@ims.u-tokyo.ac.jp (T.H.); kenishii@ims.u-tokyo.ac.jp (K.J.I.); 4 ToxOmics, NOVA Medical School (NMS), Faculdade de Ciências Médicas (FCM), Universidade Nova de Lisboa, 1099-085 Lisboa, Portugal; joao.conde@nms.unl.pt

**Keywords:** cyclodextrin-based polymer, mRNA delivery, melanoma cancer, immunotherapy

## Abstract

**Simple Summary:**

The frequency of metastatic melanoma, an extremely deadly malignancy, is rapidly increasing worldwide. Recently, messenger RNA (mRNA) injections have emerged as a promising treatment option. While mRNA therapies have demonstrated significant promise, the stability of the naked form remains a barrier, as naked mRNAs are vulnerable to common ribonucleases, and are unable to effectively penetrate plasma membranes and escape from endosomes. Thus, for this paper, we used a hyper-branched cyclodextrin-based polymer (Ppoly) as a carrier to enhance mRNA delivery for melanoma cancer. The in vitro results demonstrated that Ppoly was able to deliver the EGFP-mRNA effectively in both 2D and 3D melanoma cell lines compared to naked mRNA; in addition, Ppoly did not show any cytotoxicity. The anti-tumour effect of intratumourally injected OVA-mRNA loaded on Ppoly results showed a significant decrease in both tumour size and weight compared to other formulations by inducing an efficient adaptive immune response and OVA-specific CD8+ T cells in both spleen and tumour tissues compared to other groups.

**Abstract:**

mRNA technology has demonstrated potential for use as an effective cancer immunotherapy. However, inefficient in vivo mRNA delivery and the requirements for immune co-stimulation present major hurdles to achieving anti-tumour therapeutic efficacy. Therefore, we used a cationic hyper-branched cyclodextrin-based polymer to increase mRNA delivery in both in vitro and in vivo melanoma cancer. We found that the transfection efficacy of the mRNA-EGFP-loaded Ppoly system was significantly higher than that of lipofectamine and free mRNA in both 2D and 3D melanoma cancer cells; also, this delivery system did not show cytotoxicity. In addition, the biodistribution results revealed time-dependent and significantly higher mEGFP expression in complexes with Ppoly compared to free mRNA. We then checked the anti-tumour effect of intratumourally injected free mRNA–OVA, a foreign antigen, and loaded Ppoly; the results showed a considerable decrease in both tumour size and weight in the group treated with OVA-mRNA in loaded Ppoly compared to other formulations with an efficient adaptive immune response by dramatically increasing most leukocyte subtypes and OVA-specific CD8+ T cells in both the spleen and tumour tissues. Collectively, our findings suggest that the local delivery of cationic cyclodextrin-based polymer complexes containing foreign mRNA antigens might be a good and reliable concept for cancer immunotherapy.

## 1. Introduction

The incidence of metastatic melanoma, an extremely aggressive and fatal cancer, is steadily increasing worldwide. Early detection and the surgical removal of tumours are crucial to prevent its spread and potentially fatal outcomes. Conventional treatments like irradiation and chemotherapy have shown limited success [1]. However, new treatments focusing on oncogenic drivers have made some progress. Recently, messenger RNA (mRNA) injections have emerged as one such treatment that provides low mutational risk, good safety, and has the adaptive modularity to express a variety of diverse therapeutically useful proteins [2,3,4,5]. Research in molecular medicine is now focusing on mRNA. This is particularly true when it comes to immunisation. Many mRNA vaccines, including those for cancer immunotherapy and those against viral infections, have entered clinical trials [6,7]. The success of mRNA-based therapy has been demonstrated in numerous applications, including gene editing, protein replacement therapy, cancer immunotherapy, and immunisation against the SARS-CoV-2 virus [3,6,7]. While mRNA therapies have demonstrated significant promise, the stability of the naked form remains a barrier, as naked mRNAs are vulnerable to common ribonucleases, unable to penetrate plasma membranes effectively, and unable to escape from endosomes [8]. To overcome these obstacles, one potential solution is the encapsulation of mRNA within nanoparticles. This protective encapsulation not only shields mRNA from enzymatic degradation but also facilitates efficient cellular uptake and transport to different regions of the body. As a result, there has been a growing interest among research teams in the field of mRNA nanoparticle delivery [9,10,11]. Recent advancements in cancer immunotherapy have been attributed to the use of mRNA nanoparticles, which have demonstrated therapeutic efficacy in tumour vaccination [12,13,14]. However, a critical challenge that remains is the need for effective delivery methods. This underscores the importance of developing sophisticated and targeted delivery approaches [9,15,16]. On the other hand, polymers have attracted interest as a platform for gene delivery due to their desirable qualities, such as non-integration, scalable manufacturing, and extensive chemical structural flexibility [17,18,19]. Cyclodextrin (CD) has been recognised as an effective and efficient oligonucleotide delivery technique among polymer delivery technologies [17,18,20]. Originally employed as a plasmid DNA delivery vehicle in 1999, CD has since been recognised for its utility as a carrier for siRNAs (small interfering RNAs) [21]. CD-modified polycations have demonstrated beneficial biocompatibility and a good capacity to create stable polyplexes with therapeutic nucleic acids to keep them stable [22]. Due to their relatively high transfection effectiveness and modification adaptability, cyclodextrin-based nanosponges (CDNSs) and their derivatives have also been identified as frequently used mRNA vaccine delivery systems [23,24,25].

**Figure 1:**
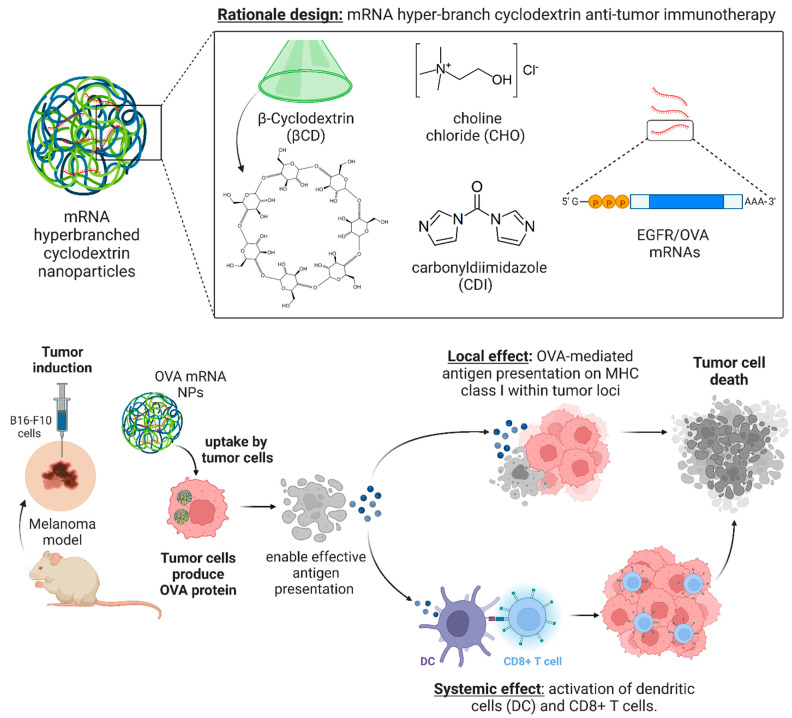
Schematic rationale of the study design.

Clinical evidence has highlighted the potential benefits of local immunotherapy in eliminating the toxicities associated with systemic treatments while promoting robust immune responses against cancer [26,27,28]. With this in mind, our investigation aims to assess whether cationic hyperbranched cyclodextrin-based polymers (Ppoly), known for their remarkable efficiency as carriers for pDNA transport in both 2D and 3D spheroid cells without toxicity [29], can effectively overcome the challenges associated with mRNA delivery for melanoma cancer in vivo. Therefore, in the present study, we aimed to investigate the capacity of our newly synthesised Ppoly by imparting positive charges to the final product using choline chloride (CHO), which has been extensively studied for its non-toxicity [30,31] and potential to be used as an effective delivery system to overcome the challenges related to mRNA delivery in cancer immunotherapy. To test this hypothesis, we pursued the following aims: Firstly, we checked the effect of Ppoly to enhance the transfection efficiency and cellular uptake of mRNA encoding the EGFP in 2D and 3D spheroid melanoma cancer cells (B16-F16). Then, the oval albumin (OVA)-mRNA, a foreign antigen, was loaded on Ppoly and administered intratumourally to pulse and induce immune cell responses in a melanoma mice model (Figure 1). The rationale behind using OVA-mRNA for this study is based on the concept of antigen presentation and the activation of the adaptive immune system. When the OVA-mRNA is translated to the OVA protein (a foreign protein) within the cancer, they can potentially present fragments of those proteins on their cell surface using molecules called major histocompatibility complex (MHC) molecules. These MHC molecules display the protein fragments to immune cells, such as T cells. If the immune system recognises the displayed protein fragments as foreign or abnormal, it can trigger an immune response against the cells displaying those fragments. This immune response can include the activation and proliferation of T cells, which can specifically recognise and eliminate cells presenting the foreign protein fragments. Furthermore, the adjuvant ability of Ppoly was tested in healthy animals.

## 2. Materials and Methods

### 2.1. Materials and Reagents

All chemical reagents were supplied by Sigma-Aldrich (St. Louis, MO, USA) without additional purification. The OVA-modified and EGFP (5 moU) mRNAs, as well as DreamFect ™ (DF40500), were provided by OZ-Biosciences (Marseille, France). Before use, β-CD and choline chloride were dried to a constant weight in an oven at 75 °C.

### 2.2. Polymer Synthesis

We followed the same steps as in our past work to create the polymer [29]. In brief, the polymer synthesis involved dissolving 1.00 g (8.81 × 10^−4^ mol) of anhydrous CD in 7.5 mL of DMSO at room temperature. Following full solubilisation, 1.11 g (7.93 × 10^−3^ mol) of choline chloride (CHO) and 1.14 g (7.05 × 10^−3^ mol) of carbonyldiimidazole (CDI) were added. In order to separate the polymer from unreacted reactants, byproducts, and solvent residues, the dry product was then dissolved in distilled water and filtered using ultrafiltration (cut-off 5 kDa). The final step was to recover the product from the ultrafiltration cell and freeze dry it to create a white powder with 22 kDa molecular weight.

### 2.3. Polymer–DNA Complex Formation and Gel Retardation Assay

Prior to the transfection procedures, a complex between cationic-CD polymers and both mRNAs (EGFP and OVA) was created. Different N/P ratios of polymer/mRNA complexes were formed (1:1, 5:1, and 10:1) by mixing 10 µL of mRNA solution (containing 1 µg of EGFP and OVA mRNA in filtered distilled water) and 10 µL of polymer solution (containing varying amounts of polymer in filtered distilled water). They were mixed, vortexed for 10 s, and then incubated for 30 min at room temperature to create the complexes. Finally, gel electrophoresis was run by mixing the loading buffer and transferring the resulting solutions onto a 1% agarose gel in 1× TAE buffer (40 mmol/L Tris acetate and 1 mmol/L EDTA) at 90 V for 45 min. DNA bands were observed using a UV trans-illuminator.

#### 2.3.1. Complexes Characterisation

As previously reported, characterisation of the complexes was carried out [29]. In brief, a 90-plus particle sizer was used to measure the size of the complexes and their zeta potential (Malvern Instruments, Malvern, UK). Furthermore, the structural characteristics and the presence of interactions between polymer and mRNA complexes were examined using scanning electron microscopy (SEM) and Fourier-transform infrared spectroscopy (FTIR), respectively.

#### 2.3.2. Determination of mRNA Encapsulation Efficiency

The ultracentrifugation approach was followed to measure the efficiency of the nanoparticle’s encapsulation of mRNA. The difference between the total amount of mRNA added to the buffer, which contains nanoparticles, and the amount of free mRNA left available in the aqueous suspension was measured via nanodrop spectrophotometry at 260 nm [32].

### 2.4. Cell Culture Protocol for 2D and 3D Spheroid Cell Culture

B16–F10 cells (passage number: 4) were obtained from the National Cell Bank of the Pasteur Institute, Tehran, Iran. The cell growth conditions were the same as in our previous study [33], where 3 × 10^4^ B16–F10 cells were used to create 3D cancer cell spheroids using the traditional hanging drop method.

#### 2.4.1. MTT (3-(4, 5-Dimethylthiazol-2-yl) 2, 5-Diphenyl Tetrazolium Bromide) Assay

The cytotoxicity of free-Ppoly at various concentrations was evaluated using the MTT assay, which was slightly modified from a previous report [34]. Furthermore, the cytotoxicity effect of different ratios (1:1, 1:5, and 1:10) of free Ppoly and complexes with mRNA was evaluated compared to lipofectamine at concentrations of 3, 6, and 12 µL in free and in complexes with mRNA after 24 h.

#### 2.4.2. Measurement of EGFP (mRNA)-Polymer Transfection Using Fluorescence Microscopy in 2D and 3D

For the 2D and 3D cell culture models, trypsinised B16–F10 cells were seeded in 12-well plates in RPMI medium with 10% FBS at a density of 1 × 10^6^ cells per well and 3 × 10^4^ cells per 20 µL drop, respectively. After 24 h, the culture medium was changed to a serum-free medium containing 1 and 2 μg of free mRNA-EGFP and complexed with PPoly at a N/P ratio of 10 for 4 h for the 2D and 3D cells, respectively. The cells were then incubated for 20 h in media containing 10% FBS. Next, PBS was used to wash the cells twice. Finally, the cells were fixed with 4% paraformaldehyde for fluorescence microscopy observation (Cytation 5 Cell Imaging Multi-Mode Reader, Agilent, Santa Clara, CA, USA), and then nuclear staining with 4′,6-diamidino-2-phenylindole (DAPI) was performed at room temperature for 30 min. In addition, the DreamFectTM Transfection Reagent (Lipofectamine), Invitrogen, Waltham, MA, USA, was used as a positive control for 24 h on both 2D and 3D cell culture, according to the manufacturer’s instructions. Furthermore, using flow cytometry, the same procedure was used to quantify the cellular uptake of free and EGFP-mRNA in complexes with PPoly at a ratio of 1:10, and Lipofectamine was used as a positive control for 24 h in both 2D and 3D cell culture at a ratio of 3:1 (Lipofectamine/EGFP). The BD FACS Calibur system was used to assess the cells and determine the fluorescence intensity of each group, which was then compared to the untreated control cells. FlowJo software was used to examine the raw data (ver. 7.6.1).

### 2.5. In Vivo Biodistribution of EGFP-mRNA Complexes with PPoly

All studies were performed in accordance with the National Research Council’s Guide for the Care and Use of Laboratory Animals. The experiments were approved by the ethical committee of Tabriz University of Medical Sciences, Pharmacy department, Iran (Ethical Code: IR.TBZMED.AEC.1402.001). The female C57BL6/J mice were 5.5 weeks old, and the tumours were established by injecting a suspension of B16–F10 cells (1 × 10^5^ cells/mL) subcutaneously (S.C.) to form the melanoma cancer [35]. Additionally, 100 μL of EGFP-mRNA (10 μg) in complex with PPoly (containing the same quantity of free mRNA) at ratio 1:10 was injected intratumourally when the tumours reached a volume of about 400–500 mm^3^ (*n* = 4 mice per group). The in vivo imaging system (Kodak live animal imaging) was used to determine the quantity of fluorescence intensity at predetermined time points.

### 2.6. Experimental Design and Tumour Induction

Female C57BL6/J mice aged six to eight weeks were obtained from the Zanjan Animal Institution in Iran. B16–F10 cells (1 × 10^5^ cells/mL) were administered subcutaneously (S.C.) into the mice to establish melanoma cancer [35]. Sixteen mice were randomly divided into four groups: untreated tumours, free-OVA mRNA, free-Ppoly, and OVA-mRNA in complexes with PPoly. When the tumours appeared, the mice were given four intra-tumoural injections of free-OVA mRNA (7 µg) in complexes with Ppoly at N/P at a ratio of 1:10, as well as the same volume of PBS for the control group. Thereafter, the mice were sacrificed after 32 days, and tumour size was measured every day using a digital calliper. At the end of the treatment period, the melanoma tumours were removed from the mice and weighed. The width, length, and height of the tumours were then measured using callipers, and the volume of the tumours was determined using the following formula: length × width 2 × 0.52 [36].

#### 2.6.1. Flow Cytometry Detection of In Vivo Immune System Cell Responses in Tumour and Spleen

After completing the treatment, the mouse tumours and spleens were aseptically removed and used to make single-cell suspensions using 70 µm filters (BD Biosciences)^67, 68^. A sharp scalpel was used to cut the tumours and spleens into pieces. The tumour and spleen tissues were quickly placed in 10 mL of the dissociation buffer (100 U/mL of collagenase IV and 100 μg/mL DNase I in RPMI media with 10% FBS), then stored at 37 °C in an incubator for 30 to 40 min. Once the 70 μm strainer was wet after adding 1 mL of cold medium to a 50 mL falcon tube, the spleens or tumours were placed on it. The organs were then lightly mashed before being passed through strainers to create single-cell suspensions. The splenocytes and tumour cells were then collected by washing the strainer with 4 mL of cold medium. The medium was aspirated off after the cells were centrifuged at 250× *g* for 5 min at room temperature. Finally, 1 mL of medium was used to resuspend the cell pellet, the purified cells were carefully collected, and the resulting suspension was washed once with FACS buffer. The cells were counted to yield 2 × 10^6^ cells per FACS tube for each sample.

#### 2.6.2. Antibodies and Flow Cytometry

The single spleen and tumours of the treated groups were incubated with antibodies in staining buffer (0.1 M PBS, 1 per cent FCS, and 0.1 per cent sodium azide) for 30 min on ice in order to stain the cell surface markers. The cells were stained with CD4 (GK1.5, PE), CD19 (eBio1D3 (1D3), PE), CD11b (M1/70, PE), and NK1.1 (PK136, FITC). Furthermore, the cells were incubated with tetramer (SIINFEKL-H2Kb APC) and a CD8a monoclonal antibody (53–6.7) (BD Biosciences) to determine tetramer+ CD8+ T cells via flow cytometry assay. The cell percentages were evaluated using FlowJo, and the graphs were made using GraphPad Prism.

#### 2.6.3. Determination of Antigen-Specific Antibodies in Serum

Using an enzyme-linked immunosorbent assay (ELISA) test, the levels of TNF-α, INF-γ and OVA-specific antibodies, including two subtypes of IgG, IgG1, and IgG2a, were measured. On day 32, serum samples were collected and kept at 4 °C. For the OVA-specific antibody procedure that followed, OVA proteins were applied to 96-well polystyrene microplates and incubated overnight at 4 °C. After serially diluting the serum samples twice in blocking buffer, they were added to the washed microplates. The prediluted horseradish peroxidase-conjugated goat and anti-mouse antibodies IgG, IgG1, or IgG2a (Abcam, Waltham, MA, USA) were added to the microplates after 2 h incubation at 37 °C. Tetramethylbenzidine substrate was added after an additional two hours of incubation, and the chromogenic process was subsequently inhibited by 2MH_2_SO_4_. Thereafter, a microplate reader assessed each well’s optical absorbance at 450 nm (Tecan, Männedorf, Switzerland).

#### 2.6.4. Histopathological Studies

Neutral formaldehyde (10 per cent) was used to fix the removed tumours. The samples were dehydrated using various concentrations of ethanol (30%, 50%, 70%, 90%, and 100%), after which the tissues were cleaned in benzene and embedded in low melting-point paraffin wax. Thereafter, 5 µm thick sections were mechanically cut into sections and sequentially placed on clean glass slides. These slides were then stained with Hematoxylin and Eosin for examination under a light microscope (LEICA DM 3000, Leica, Shanghai, China).

#### 2.6.5. Assessment of the Adjuvanticity of Ppoly

The experiments were conducted in accordance with the appropriate laws and with the approval of the Animal Experiment Committee of the Institute of Medical Science, University of Tokyo. Six-week-old female C57BL6/J mice were obtained from CLEA, Japan. The OVA protein (10 μg) was mixed with Ppoly (35 μg) in 20 μL of PBS. The mice were then injected with the sample solutions intramuscularly on days 0 and 14. On day 28, the serum was collected from the immunised mice. OVA-specific antibodies were measured as mentioned above by using horseradish peroxidase-conjugated anti-mouse IgG, IgG1, IgG2c, and IgE antibodies (Southern Biotech, Birmingham, AL, USA). Titers of the OVA-specific antibodies were determined via log-linear interpolation of the serum dilution value corresponding to the cut-off absorbance (OD450 of 0.2).

### 2.7. Statistical Analysis

Statistical analysis was conducted using GraphPad Prism 8 (GraphPad Software, Inc., La Jolla, CA, USA). The data were analysed using one-way ANOVA (Analysis of Variance). All samples were analysed in triplicate and are presented as mean ± standard deviation (SD) for *n* = 3–5. The significance level was calculated by the *p*-value. Statistically, a *p* value < 0.05 was considered significant.

## 3. Results and Discussion

### 3.1. Design Rationale and Characterisation of mRNA Nanoparticles

**Figure 2:**
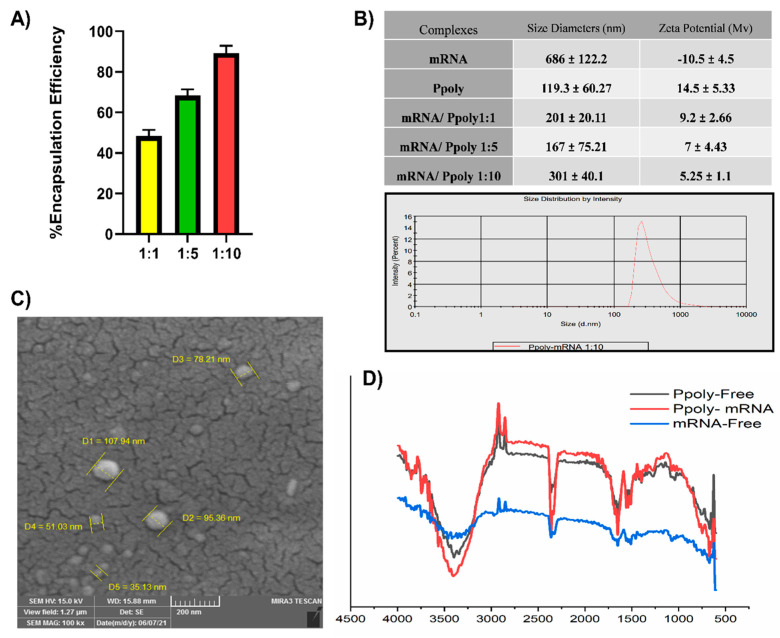
(**A**) Encapsulation efficiency of Ppoly for mRNA. (**B**) Size of Ppoly/mRNA complexes at different N/P ratios and zeta-potential of DLS. (**C**) SEM image of Ppoly/mRNA at an N/P ratio of 10:1. (**D**) The FTIR spectra of PPoly free, mRNA free and Ppoly/mRNA complexes. The results are expressed as means ± SD.

Our specific goal and the basis of our overall hypothesis was to develop an effective anti-tumour immunotherapy via the local delivery of mRNA loaded on hyper-branched cyclodextrin nanoparticles. To be considered a successful gene delivery vehicle, the vector must be able to condense nucleic acids [37]. To confirm the ability of the synthesised polymer nanoparticles to interact electrostatically with negatively charged mRNA to produce Ppoly/mRNA complexes, several functional assays, such as electrophoretic mobility shift assay (EMSA), particle size analysis, zeta potential analysis, and SEM imaging, were performed [38]. To evaluate the condensation efficiency of Ppoly with mRNA, an electrophoretic mobility shift assay was performed [39]. As can be seen in Figure S1A, even at the lowest N/P ratio, both EGFP (right) and OVA (left) mRNAs were almost completely retained in the wells by Ppoly; however, interestingly, the N/P ration 10:1 showed the highest encapsulating efficiency (EE), about 90% compared to 55% at 1:1 (Figure 2A), which demonstrated improved complex formation between the polymer and mRNAs through ionic interactions. Furthermore, as displayed in Figure 2B, when the Ppoly/mRNA weight ratio reached 10:1, the Ppoly could successfully condense mRNAs into a particle size of 300 nm with a positive potential (about +5 mV). Therefore, the particle size and shape of the Ppoly/mRNA complex at that ratio were then further examined by SEM and FTIR (Figure 2C,D). The SEM image demonstrated that the Ppoly/mRNA complexes were spherical in shape. Additionally, the 10:1 N/P ratio FTIR spectrum showed that mRNA complexes were successfully incorporated into the Ppoly (Figure 2D).

### 3.2. Two-Dimensional (2D) and Three-Dimensional (3D) Spheroid Cytotoxicity and Uptake Analysis

**Figure 3:**
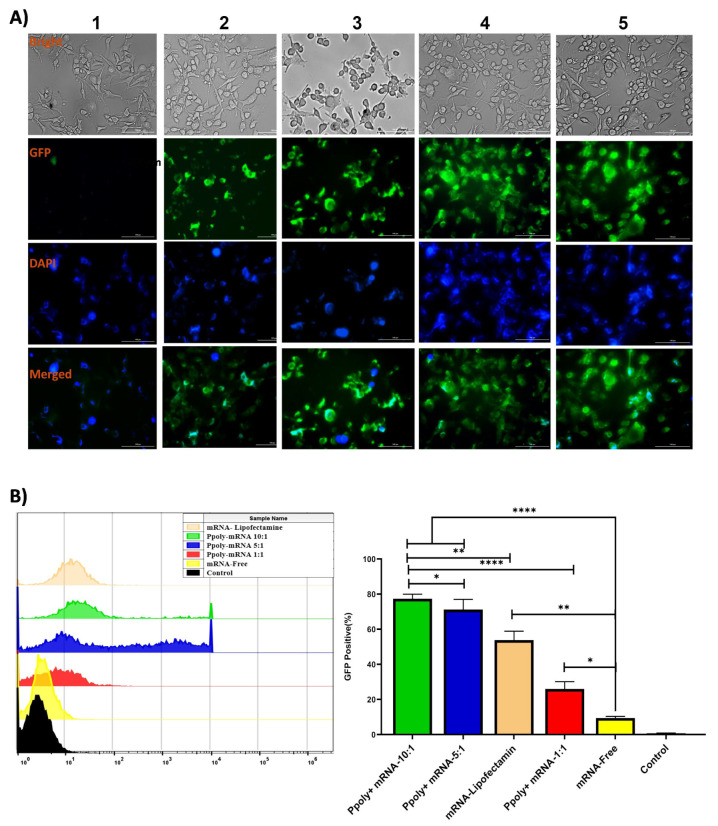
(**A**) Fluorescence microscopy results for the free EGFP-mRNA and EGFP-mRNA in complexes with Lipofectamine and Ppoly. **1**—Free mRNA, **2**—Lipofectamine, **3**—1:1 (mRNA/Ppoly), **4**—1:5, **5**—1:10. (**B**) Flow cytometry results for the free EGFP-mRNA and EGFP-mRNA in complexes with Lipofectamine and CD-NSs (* *p* < 0.05, ** *p* < 0.01, **** *p* < 0.0001).

The cytotoxicity effect of different ratios—1:1, 1:5, and 1:10—of Ppoly free and complexes with mRNA compared to a commercial lipofectamine agent at concentrations of 3, 6, and 12 µL (routine amount used for gene delivery) in free and in complex with mRNA was assessed in melanoma cancer cell lines (B16–F10). The results illustrated that Ppoly at different ratios did not show cytotoxicity, while lipofectamine at 6 and 12 µL showed about 32% and 52% toxicity, respectively (Figure S2A–C). These findings underscore the advantages of utilizing modified cationic polymers, such as Ppoly, in terms of their enhanced safety and effectiveness. This could potentially overcome existing delivery barriers and prove beneficial for various in vivo applications. By minimizing cytotoxic effects, Ppoly offers a more favourable profile for in vivo applications, ensuring the viability and integrity of targeted cells [34,35]. Based on the characterisation results, formulations with N/P ratios of 1:1, 1:5, and 1:10 were chosen for the 2D cellular transfection study to compare the ability of the Ppoly positive charge to deliver the EGFP-mRNA inside the B16–F10 cells to free mRNA and mRNA in complex with the commercial lipofectamine agent using fluorescence microscopy and flow cytometry. Strong green fluorescence related to EGFP protein expression was detected in cells treated with mRNA complexes with Ppoly at ratios of 1:5 and 1:10 compared to lipofectamine (1:3 ratio) and also free mRNA. It should be emphasised that the intensity of green fluorescence within the cells reflects the capacity of the cells to uptake our NPs (Figure 3A). In addition, the quantitative uptake of complexes containing mRNA-EGFP at different N/P ratios was assessed by using a flow cytometer after incubation for a certain time, and the uptake efficacy was found to be about 77%, 72%, 26%, and 58% for the mRNA in complex with Ppoly 1:10, 1:5, 1:1, and lipofectamine, respectively (Figure 3B), while the naked mRNA showed a negligible level of EGFP expression due to the inefficiency of the cellular entry. These findings, which were in line with publications in the literature [39,40,41,42], indicated that Ppoly loaded with mRNA enhanced transfection efficiency by postponing the release of mRNA from nanoparticles compared to free mRNA.

**Figure 4:**
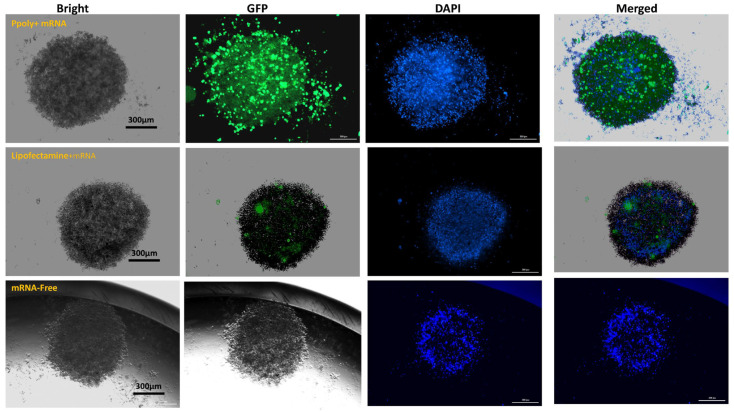
Microscopic fluorescence images of 3D spheroids (B16–F10 cells), mRNA/Ppoly nanocomplexes transfection at N/P ratio 10:1 compared to free mRNA-free, and lipofectamine after 24 h of incubation.

Three-dimensional (3D) cell culture has gained popularity due to its ability to simulate living cells within micro-assembled supports and devices with a 3D structure tailored to the microarchitecture of a tissue or organ. Therefore, the ability of the designed mRNA NPs to enter a 3D spheroid structure was also assessed. To evaluate the uptake and transfection of the mRNA-loaded Ppoly compared to free mRNA, spheroids of B16–F10 cells were created. The outcomes showed that, after 24 h of incubation with the spheroid cells, free mRNA was unable to be taken up by 3D cancer cells (Figure 4), whereas EGFP expression in 3D spheroid cells transfected by Ppoly-mRNA complexes at that time was significantly higher than lipofectamine and free mRNA (Figure 4). It is interesting to note that Ppoly loaded with EGFP-mRNA demonstrated high transfection efficiency in 2D monolayer cells (B16–F10), while these values in 3D spheroids were lower, as demonstrated by other studies on gene delivery in 3D spheroids, which found similarly low transfection efficiency [43,44,45,46]. The extracellular matrix (ECM) and high-cell-density multicellular barriers in the deep regions of 3D spheroids are the main barriers preventing gene delivery agents from penetrating the spheroids’ cells. These cells are known to have extensive cell contact, increase interstitial pressure simultaneously, and display significant resistance to chemotherapy and radiation therapy [47]. We noticed this phenomenon in our study and demonstrated that it holds true for transfection in both two-dimensional and three-dimensional spheroids. The outer cell layers in 3D spheroids were the only areas of the peripheral cells that were transfected by Ppoly/mRNA complexes (N/P ratio 10:1), which could explain this phenomenon. Furthermore, these findings support those of other studies using the in vitro 3D spheroid model for gene delivery [46,48], which produced clinical findings indicating poor non-viral gene transfer effectiveness in vivo in tissues with three-dimensional structures or solid malignancies.

### 3.3. In Vivo Immunotherapeutic Efficacy of mRNA Nanocomplexes

**Figure 5:**
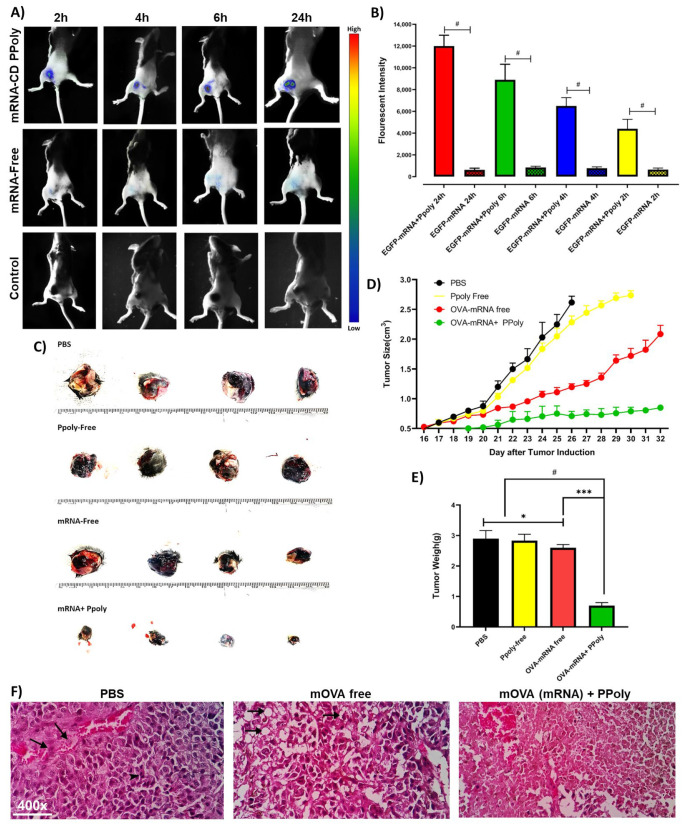
(**A**) Typical in vivo images of female C57BL6/J mice, inratumoural injection of saline (control group), free mRNA, and mRNA in complex with Ppoly all at a dosage of 10 µg EGFP-mRNA. (**B**) Quantitative assay of fluorescence intensity from the tumours of different groups based on in vivo images. (**C**) The anti-tumour growth effect of OVA-mRNA both free and in complexes with positively charged Ppoly. (**D**) The mice of the PBS, OVA-mRNA, free Ppoly, and OVA-mRNA + Ppoly groups and their excised tumours were imaged at day 25 and 32 post-tumour induction, respectively. (**E**) Tumour measurements were performed daily using callipers, and the average tumour volume was calculated as length × width^2^ × 0.52 to represent the average of tumour weight and statistical size differences among the groups. (Error bars represent the SD, and significance was determined using One-Way ANOVA (ns, Not Significant; * *p* < 0.05, *** *p* < 0.001, # *p* < 0.0001)). (**F**) The microscopic appearance of tumour mass from the group treated with “PBS”. The tumour cells were arranged in nests or as cords around the blood vessels and mitotic figures (arrows) exhibiting a remarkably small blood supply with the invasion of tumour cells into the blood vessels (arrows) and mitotic figures (arrow heads). The OVA-mRNA-treated group indicated extensive tumour cell vacuolation (arrows), swelling, rupture, and fragmentation, as well as a prominent decrease in cell population; the neoplastic tissue was rather degenerated, and characteristics of degeneration were more prominent with the decrease in cell population. Furthermore, the OVA-mRNA +Ppoly-treated group’s tumour tissues were rather necrotic, with characteristics of coagulative necrosis with the massive necrosis of melanoma cells (H&E, 400×).

Next, we studied the in vivo pharmacokinetics and therapeutic efficacy of the Ppoly/mRNA complexes in a melanoma mouse model. Melanoma cases are rising across the world [49]. Meanwhile, the death rate from melanoma has decreased due to improvements in the management of systemic diseases with immunotherapy and targeted therapies. However, survival can present several problems, such as second primary melanomas, an increased chance of developing other skin cancers, and the long-term effects of melanoma therapy [50,51]. As a result, there is an urgent need to develop new immunotherapies. To evaluate the in vivo delivery efficacy of mRNA in complexes with Ppoly, free EGFP-mRNA (10 µg) and EGFP-mRNA in complex with Ppoly at a ratio of 1:10 were injected intratumourally into female C57BL6/J melanoma cancer mice. To evaluate the ability of EGFP-mRNA-loaded Ppoly expression in a melanoma tumour site, the fluorescence intensity was observed at 2, 4, 6, and 24 h after intratumoural injection through an in vivo imaging system (Kodak live animal imaging) with an excitation wavelength of 480 nm and an emission wavelength of 510 nm (Figure 5A). The results showed that the fluorescence intensity in the group receiving EGFP-mRNA-Ppoly was significantly increased in a time-dependent manner compared to free mRNA; a group receiving a saline solution was considered the control group (Figure 5B). This suggests that Ppoly could be considered an efficient local delivery system due to its ability to concentrate and deliver the mRNA to the tumour, which is consistent with the findings of other studies [52,53,54]. Therefore, we then assessed the in vivo anti-cancer activity of OVA-mRNA free and in complexes with Ppoly in an induced melanoma animal tumour model. C57BL/6 mice were treated four times in four-day intervals once the melanoma tumours were palpable (*n* = 4 mice). When the tumours became visible and palpable, the size was measured every other day using a vernier calliper. Mice treated with PBS and Ppoly-free formulations developed extensive tumours. In contrast, those treated with OVA-mRNA exhibited significantly slower and negligible tumour growth. Impressively, the complexes of OVA-mRNA with Ppoly demonstrated approximately three times more tumour suppression compared to the untreated group, indicating the potential of OVA-mRNA-Ppoly therapy in preventing the growth of malignant tumours. Interestingly, there were no significant differences in average tumour weight between the PBS and free-Ppoly groups (Figure 5C). Interestingly, there were no differences in average tumour weight between PBS and free-Ppoly, while the average tumour weight in the Ppoly-OVA-mRNA group was significantly lower than in the other groups (Figure 5D,E). Notably, the mice in the PBS groups had to be sacrificed by day 25 due to the rapid expansion and excessive size of the tumours. To understand the change in tumour tissue morphology in the treated groups, they were further checked via histopathological examination. The microscopic morphology of the group treated with PBS exhibited solid sheaths of neoplastic cells with fairly vascular, congested blood vessels, arranged in irregular cords around a blood vessel, and the nuclei of neoplastic cells were large, ranging in shape from ovoid to round to irregular polygons with high mitotic indexes (Figure 5F). In the mRNA treatment group, a proportionate increase in vacuolar degeneration and the necrotic areas of the tumour was observed along with the characteristics of degeneration, which include swelling, vacuolisation, rupture, and fragmentation. In addition, the cytoplasm of degenerating melanoma cells contained varying amounts of fine, light brown melanin pigment. Mitotic figures were significantly decreased compared to the untreated group, as the malignant cell population was lower in this group with a remarkably small blood supply. In contrast, in the group treated with OVA-mRNA loaded Ppoly, the parenchyma of the tumour mass primarily consisted of necrotic areas containing tumour cells with pyknotic, karyorrhectic nuclei, and eosinophilic cytoplasm.

#### Cellular and Humoral Immune System Responses to mRNA Therapy

**Figure 6:**
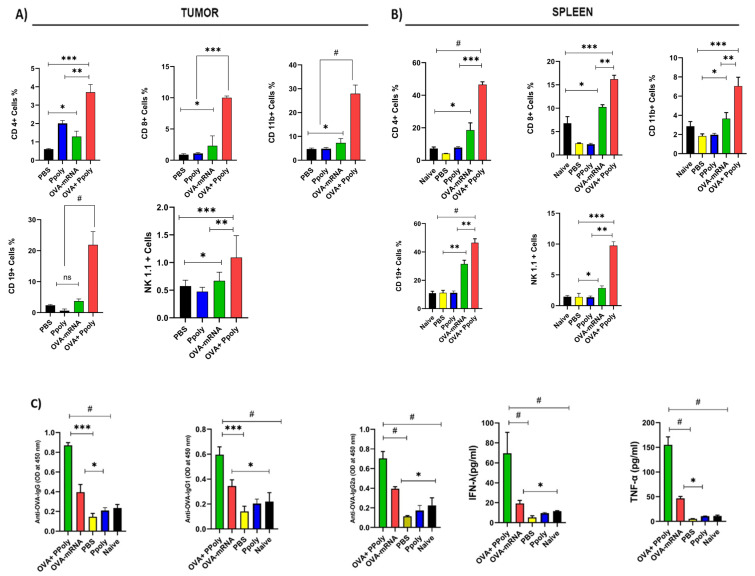
The cellular immune system response in C57BL/6 mice tumour and spleen tissues. On the final day of the treatment period, (**A**) the tumours and (**B**) the spleens were excised and homogenised, and immune cell infiltration was analysed via flow cytometry. Antigen-specific antibodies induced by OVA-mRNA +Ppoly nanocomplexes. (**C**) After the end of the treatment period, the OVA-specific antibodies, including serum IgG antibodies, IgG1 antibodies, and IgG2a antibodies, were examined in the serum. In addition, the serum level of TNF-α and IFN-γ was assessed in different groups. (Error bars represent the SD, and significance was determined using One-Way ANOVA (ns, Not Significant; * *p* < 0.05, ** *p* < 0.01, *** *p* < 0.001, # *p* < 0.0001).

The initial activation of adaptive immune activity is necessary for the formation of efficient and long-lasting anti-tumour immunity [55,56,57]. So, we assessed the variations in the immune cell populations of both humoral and cellular immunity using isolated splenocytes and single-tumour cells to confirm whether the effective anti-tumour effect of OVA-mRNA-loaded Ppoly was due to an endogenous host immune cell response. For this, firstly, the harvested tumour and spleen single cells were stained with anti-CD8a (cytotoxic T cells), CD4 (T- helpers), CD19 (B cells), CD11b (macrophages), and NK1.1 (natural killer). The results showed that mice given OVA-mRNA-Ppoly complexes had higher percentages of almost all subtypes of leukocytes in the tumour (Figure 6A) and spleen mononuclear cells (Figure 6B), including B lymphocytes, macrophages, T-helpers, CTL, and NK cells compared to the OVA-mRNA-free and control groups, which is consistent with the results of other studies on the effects of mRNA vaccines designed to stimulate cellular and humoral immune responses against foreign antigens [58,59].

**Figure 7:**
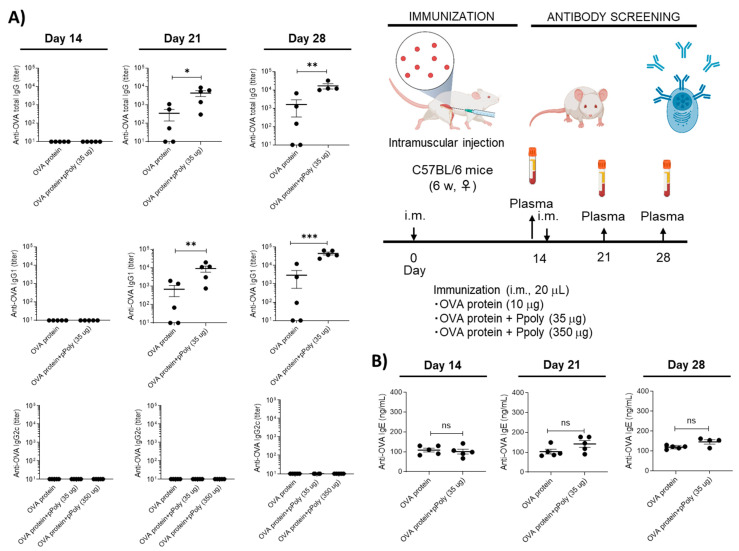
(**A**) The humoral immune system response of immunised mice treated with free OVA protein (10 µg) and OVA protein in complexes with Ppoly (35 µg) by evaluating OVA-specific antibodies, including serum IgG antibodies, IgG1 antibodies, and IgG2a antibodies, in the plasma. (**B**) The IgE production by immunised mice treated with free OVA protein (10 µg) and the OVA protein in complexes with Ppoly (35 µg). (ns, Not Significant; * *p* < 0.05, ** *p* < 0.01, *** *p* < 0.001).

The mRNA molecules reach the cytoplasm after vaccination, where they are translated into proteins. Cytosolic proteasomes break down dendritic cells (DC), producing proteins, and the resultant epitope peptides are then delivered via the MHC class I pathway, which triggers an antigen-specific CD8+ T cell response [60]. Moreover, the uptake of the extracellular antigens by DCs will induce a CD4+ helper T cell response [61]. Therefore, we hypothesised that the OVA mRNA is translated into OVA protein, where it is processed by the immunoproteasome, which is then processed to enable effective antigen presentation by the antigen-presenting cells (APC) to CD8+ T lymphocytes [57]. Thus, we quantified the OVA antigen in tumour and spleen cells, which consist of a variety of immune cell populations [62,63] excised from each group of the treated mice using flow cytometry. Interestingly, mice treated with the OVA-mRNA-Ppoly complex had expressed approximately 11% MHC class I -OVA peptides in tumour cells (Figure S1B) and 4.5% in spleen cells, two times more than free-OVA. This suggests that the Ppoly can deliver the mRNA efficiently and that it enhances antigen presentation by MHC class I peptides at the site of tumour cells. Furthermore, the expansion of OVA-specific CD8+ T cells was evaluated using tetramer+ (SIINFEKL-H2Kb APC) and CD8a for the detection of H-2Kb/SIINFEKL (OVA)-specific CD8-T+ cells [55]. As shown in Figure S1B,C, compared with untreated mice, OVA-mRNA complexes with Ppoly resulted in a significant increase in CD8+Tetramer+ T cells in tumour and spleen mononuclear cells (0.023% vs. 0.8% and 0.010% vs. 0.4%, respectively). The increased potency of OVAmRNA complexes with Ppoly compared to OVAmRNA alone was abundantly supported by tumour growth data. The suppression of tumour growth in the OVA-mRNA-Ppoly-treated group can be attributed to the increased number of OVA-specific CD8+ T cells. This elevated presence of CD8+ T cells enhances the recognition of specific tumour cell antigens by cytotoxic T lymphocytes (CTLs). Consequently, the CTLs release cytotoxic molecules such as perforin and granzymes into the tumour cells, inducing cellular damage. This process activates caspases, which are enzymes responsible for initiating the apoptotic cascade, leading to the death of the tumour cells. Furthermore, previous studies have indicated that this mechanism also promotes the proliferation of CTLs, contributing to the overall suppression of tumour growth [15,64,65]. Moreover, serum samples were collected, and the levels of induced OVA-specific total immunoglobulin G (IgG) and IgG subclasses (IgG1 and IgG2a) were measured to confirm the humoral immune responses to various formulations. According to the results shown in Figure 6C, OVA-mRNA-Ppoly significantly increased the levels of total IgG, IgG1, and IgG2a compared to naked mRNA. Free Ppoly did not induce humoral responses, confirming that Ppoly alone cannot induce antigen-specific immune responses. Notably, a naked mRNA injection could produce moderate humoral immune responses. This effect was observed previously, and investigations have since demonstrated that various humoral responses can be partially induced by mRNA injection [55]. These results suggest that Ppoly formulation could indeed enhance the cellular delivery of mRNA and result in stronger humoral immune responses, which may lead to an increase in antibody-dependent cell-mediated cytotoxicity. Both successful antigen presentation and appropriate immunostimulatory signals must be produced for immunotherapy to be effective. Therefore, the serum levels of proinflammatory cytokines were assessed to confirm the immune-stimulating effect of OVA-mRNA-Ppoly. The serum levels of the tumour necrosis factor (TNF-α) and interferon gamma (IFN-γ), which are important markers of a strong immune response, were measured using an enzyme-linked immunosorbent assay (ELISA). Interestingly, when compared to the untreated and naked mRNA-treated groups, the OVA-mRNA-Ppoly complex induced higher IFN-γ and TNF-α levels. This highlighted the synergistic immune-stimulating effects of Ppoly-encapsulating mRNA (Figure 6C). These findings demonstrate that, in comparison to mRNA alone, OVA-mRNA-Ppoly could boost cellular immune responses. It has been shown that TNF-α secretion mainly leads to increased apoptosis and inflammation, while IFN-γ secretion leads to an increase in CTL cell activity and Th1 differentiation, which in turn can activate macrophages in a classical pathway to increase inflammation in targeted cells [66]. Therefore, both the strong stimulation of cellular and humoral immune system responses and high-level secretion of these cytokines could partially explain the antitumor effect of OV-mRNA loaded on Ppoly. To further verify the robust immunostimulatory effect mechanism of the mRNA–Ppoly complex, we examined whether Ppoly possesses adjuvanticity by intramuscularly immunising the normal mice with a mixture of the OVA protein and Ppoly. As a result, the production of OVA-specific IgG1 was increased in the presence of Ppoly, although there was no change in that of OVA-specific IgG2c (Figure 7A). The levels of antigen-specific IgG1 and IgG2c reflect the induction of Th2 and Th1 responses, respectively [67]. Therefore, these data suggest that Ppoly can moderately increase the antigenicity of the loaded OVA protein compared to free OVA via an increased induction of Th2-related immune responses.

Given that many vaccinations are intended to be administered to healthy individuals, one of the most crucial qualities of vaccine adjuvants, in addition to their efficiency, is their safety. Traditionally, it has been considered that Th2 and IgE (an allergic antibody) responses are sequential [68]; therefore, we checked the IgE secretion levels in mice treated with the OVA protein (both free and in complexes with Ppoly) because the ability of an adjuvant to induce IgE may be a crucial risk factor affecting the allergenic potential of vaccines. The data suggested that Th2 induction does not always induce strong IgE production because Ppoly at defined concentrations did not lead to the production of IgE in mice (Figure 7B). This result was consistent with the findings of a study by Hayashi et al., which demonstrated the effective adjuvant ability of Hydroxypropyl-β-Cyclodextrin complexes with the OVA protein [69,70]. As a result, these data suggest that Ppoly in complexes with the OVA protein could induce Th2-related immune responses in a safety pathway, while the mRNA–Ppoly complex induced not only Th2 but also Th1 responses, such as increased CD8^+^ T cells, antigen-specific IgG2a, and IFN-γ production (Figure 6A–C). Collectively, complexation between therapeutical mRNA and Ppoly may lead to the robust induction of cellular immune responses including the Th1, Th2, and CTLs, which are mainly expected to contribute to immunotherapeutic effects in the tumour model.

## 4. Conclusions

The foundations were laid for the rapid development of mRNA vaccinations during the COVID-19 pandemic by years of research into mRNA vaccines for cancer treatment in preclinical and clinical trials. The inherent benefit of ease of production, which rivals the best conventional vaccine manufacturing methods currently available, makes therapeutic cancer vaccines based on mRNA an attractive option for cancer immunotherapy. These vaccines are well-tolerated and have the potential to be a powerful tool in the fight against cancer [71].

In this study, we conducted a comprehensive evaluation of the efficacy and safety of Ppoly as an mRNA delivery vehicle. Initially, we demonstrated that Ppoly efficiently delivered EGFP-mRNA to both 2D and 3D melanoma cell lines, surpassing the delivery efficiency of naked mRNA. Importantly, we observed no cytotoxic effects associated with Ppoly, further establishing its safety as a delivery system. These findings underscored the potential of Ppoly as a robust and secure mRNA delivery vehicle, as confirmed by in vitro cytotoxicity assays.

Building upon these promising results, we proceeded to evaluate the biodistribution of mRNA-EGFP following intratumoural injection in mice with induced melanoma cancer. The time-dependent analysis revealed that EGFP expression was higher and more sustained over time when delivered in complexes with Ppoly compared to free EGFP.

Next, we investigated the anti-tumour effects of intratumourally injected OVA-mRNA loaded on Ppoly. The results showed a significant decrease in both tumour size and weight in the group treated with OVA-mRNA in complexes with Ppoly compared to other formulations. Furthermore, we sought to understand the immunological mechanisms underlying the observed anti-tumour effects. We discovered that OVA-mRNA, as a foreign antigen, complexed with Ppoly, elicited a robust adaptive immune response in vivo. This was evidenced by the substantial increase in most leukocyte subtypes and the remarkable expansion of OVA-specific CD8+ T cells observed in both spleen and tumour tissues, surpassing the immune responses observed in untreated groups. Additionally, OVA-mRNA, in combination with Pploy, significantly improved humoral immune responses in mice with induced melanoma tumours. These facts were considered in the new immunotherapy strategy, which resulted in efficient anti-tumour immunity against melanoma cancer and significantly inhibited tumour growth. The host immune system detects tumour cells that display non-self-foreign antigens as foreign or infected cells [54], which is the reason why the delivery of foreign antigens via polymeric complexes can have anti-tumour effects. The results of this work demonstrate that the intra-tumoural administration of a cationic hyper-branch cyclodextrin-based polymer containing a foreign antigen—OVA-mRNA—may be a new and promising therapy to promote the immunological treatment of melanoma cancer, with a moderately adjuvant ability to induce Th2-related immune responses in a safety pathway. However, more in-depth studies are required to better understand the delivery mechanism and enhance the efficiency of the mRNA inside this polymer; comparisons between this system and other existing carriers are also required.

## Data Availability

The data presented in this study are available in this article (and Supplementary Materials).

## Supplementary Materials

The following supporting information can be downloaded at: https://www.mdpi.com/article/10.3390/cancers15143748/s1. Figure S1. (A) Gel retardation assay for OVA, left, and EGFP-mRNA, right. (B) Quantification of H2-Kb SIINFEKL expression and SIINFEKL+ CD8+ T cells antigen specific T cell responses in tumor tissues, and (C) Spleen tissues following treatment with formulations. Figure S2. (A) Cytotoxicity results for different concentrations of Ppoly. (B) Different ratios (1:1, 1:5 and 1:10) of Ppoly free and complexes with mRNA. (C) Different volumes of Lipofectamine (µL) free and complexes with mRNA against melanoma cancer cell lines.
