# Supplementary material for: Intratumoural Delivery of mRNA Loaded on a Cationic Hyper-Branched Cyclodextrin-Based Polymer Induced an Anti-Tumour Immunological Response in Melanoma
Source: Cancers (Basel). 2023 Jul 24;15(14):3748. doi: 10.3390/cancers15143748 (PMC10378402; doi:10.3390/cancers15143748)
Supplement: Supplementary file 1 [file cancers-15-03748-s001.zip › cancers-2495072-supplementary.pdf]

Supplementary information

Intratumoural Delivery of mRNA Loaded on a Cationic Hyper-Branched Cyclodextrin-Based Polymer Induced an Anti-Tumour Immunological Response in Melanoma

Yousef Khazaei Monfared [1,†], Mohammad Mahmoudian [2], Parvin Zakeri-Milani [2,*], Claudio Cecone [1], Tomoya Hayashi [3], Ken J. Ishii [3], João Conde [4], Adrián Matencio [1] and Francesco Trotta [1,*,†]

[1] Department of Chemistry, University of Turin, 10125 Turin, Italy; yousef.khazaeimonfared@unito.it (Y.K.M.); claudio.cecone@unito.it (C.C.); adrian.matencioduran@unito.it (A.M.)

[2] Faculty of Pharmacy, Tabriz University of Medical Sciences, Tabriz 5165665931, Iran; mahmoodian.nano@gmail.com

[3] Division of Vaccine Science, Department of Microbiology and Immunology, The Institute of Medical Science, The University of Tokyo (IMSUT), Tokyo 113-8654, Japan; tomoya-h@ims.u-tokyo.ac.jp (T.H.); kenishii@ims.u-tokyo.ac.jp (K.J.I.)

[4] ToxOmics, NOVA Medical School (NMS), Faculdade de Ciências Médicas (FCM), Universidade Nova de Lisboa, 1099-085 Lisboa, Portugal; joao.conde@nms.unl.pt

* Correspondence: pzakeri@tbzmed.ac.ir (P.Z.-M.); francesco.trotta@unito.it (F.T.)

† These authors contributed equally to this work

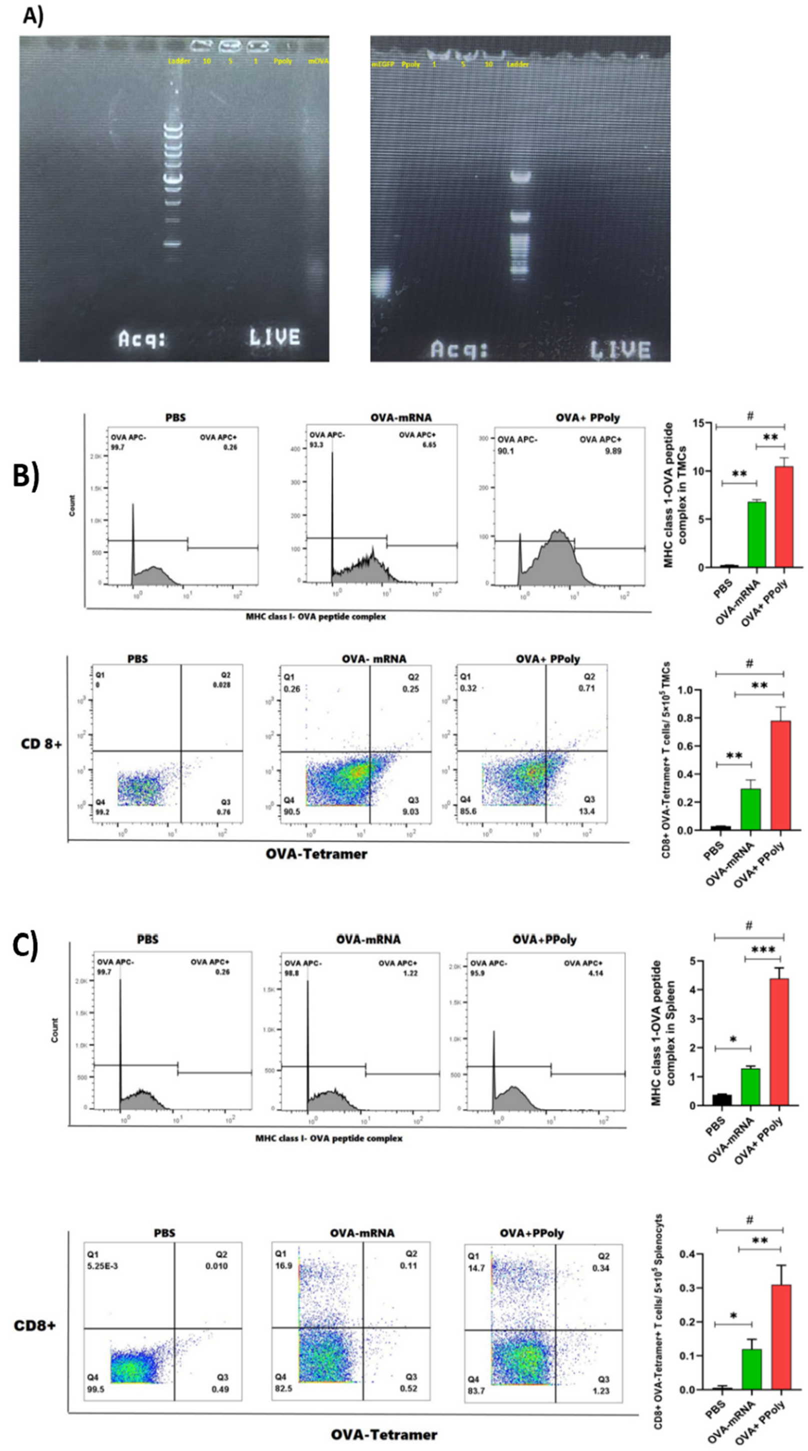

Figure S1. **A,** Gel retardation assay for OVA, left, and EGFP-mRNA, right. **B,** Quantification of H2-Kb SIINFEKL expression and SIINFEKL+ CD8+ T cells antigen specific T cell responses in tumor tissues, and **C,** Spleen tissues following treatment with formulations. (* $p < 0.05$, ** $p < 0.01$, *** $p < 0.001$, # $p < 0.0001$).

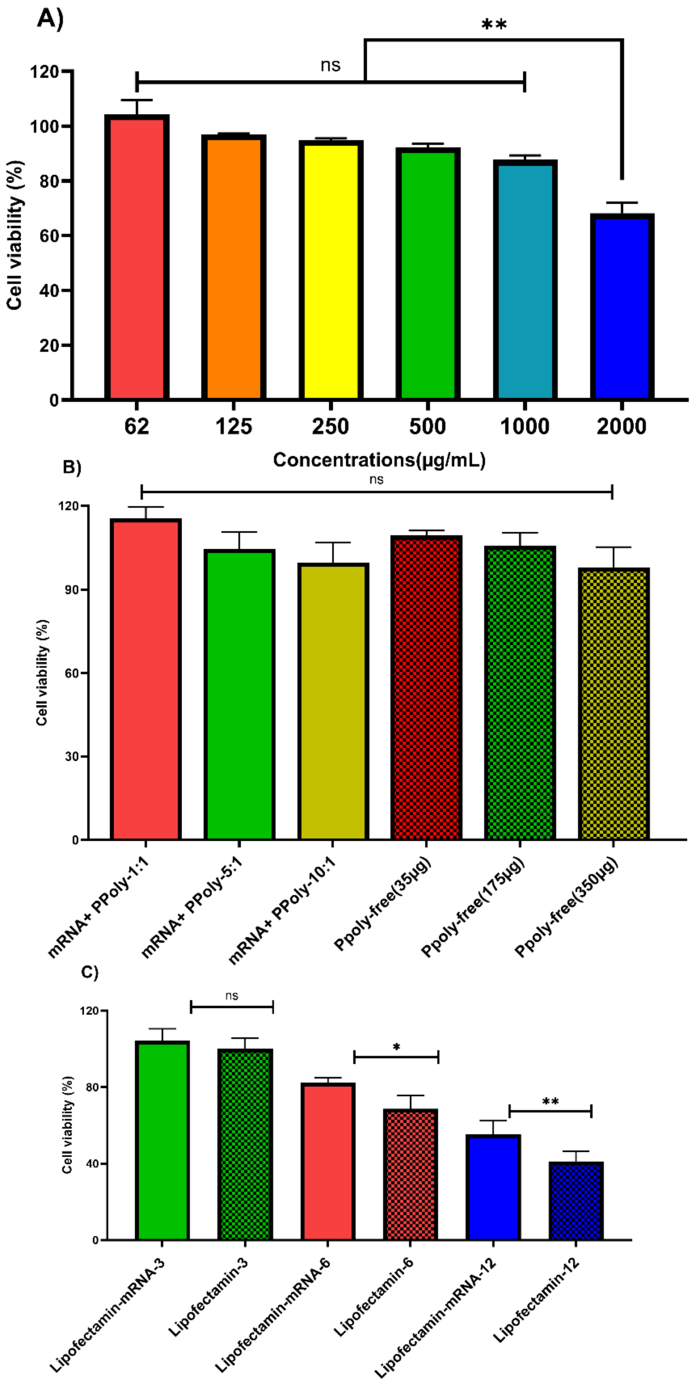

Figure S2. **A**, Cytotoxicity results for different concentrations of Ppoly. **B**, Different ratios (1:1, 1:5 and 1:10) of Ppoly free and complexes with mRNA. **C**, Different volumes of Lipofectamine (µL) free and complexes with mRNA against melanoma cancer cell lines. (ns, Not Significant; * $p < 0.05$, ** $p < 0.01$)
